# Comparative Analysis of LiDAR-SLAM Systems: A Study of a Motorized Optomechanical LiDAR and an MEMS Scanner LiDAR

**DOI:** 10.3390/s25175352

**Published:** 2025-08-29

**Authors:** Simone Fortuna, Sebastiano Chiodini, Andrea Valmorbida, Marco Pertile

**Affiliations:** 1 CISAS “Giuseppe Colombo”, University of Padova, Via Venezia 1, 35131 Padova, Italy; simone.fortuna@phd.unipd.it (S.F.); sebastiano.chiodini@unipd.it (S.C.); andrea.valmorbida@unipd.it (A.V.); 2 Department of Industrial Engineering (DII), University of Padova, Via Venezia 1, 35131 Padova, Italy

**Keywords:** FAST-LIO 2, LiDAR, navigation, robotics, SLAM

## Abstract

Simultaneous Localization and Mapping (SLAM) is crucial for the safe navigation of autonomous systems. Its accuracy is not based solely on the robustness of the algorithm employed or the metrological performances of the sensor, but rather on a combination of both factors. In this work, we present a comprehensive comparison framework for evaluating LiDAR-SLAM systems, focusing on key performance indicators including absolute trajectory error, uncertainty, number of tracked features, and computational time. Our case study compares two LiDAR-inertial SLAM configurations: one based on a motorized optomechanical scanner (the Ouster OS1) with a 360° field of view and the other based on MEMS scanners (the Livox Horizon) with a limited field of view and a non-repetitive scanning pattern. The sensors were mounted on a UGV for the experiments, where data were collected by driving the UGV along a predefined path at different speeds and angles. Despite substantial differences in field of view, detection range, and noise, both systems demonstrated comparable trajectory estimation performance, with average absolute trajectory errors of 0.25 m for the Livox-based system and 0.24 m for the Ouster-based system. These findings underscore the importance of sensor–algorithm co-design and demonstrate that even cost-effective, lower-field-of-view solutions can deliver competitive SLAM performance in real-world conditions.

## 1. Introduction

The growing demand for autonomous navigation in GNSS-denied and unstructured environments has stimulated significant advancements in LiDAR sensor technology characterized by diverse capabilities and specifications [1]. Within the range of available scanning mechanisms, mechanical spinning LiDARs remain widely used in the automotive sector due to their proven reliability. Livox LiDAR sensors [2], offering a favorable trade-off between cost efficiency and measurement precision, are similarly employed across a broad range of applications. Nevertheless, in comparison to traditional spinning LiDAR systems, Livox devices are constrained by a narrower field of view, which may limit the quantity of environmental features detected in each scan. Despite advances in LiDAR technology, reliable perception alone is not sufficient for autonomous operation. An essential component for autonomous systems, including rovers on planetary exploration missions, is Simultaneous Localization and Mapping (SLAM) [3], as it allows the rover to navigate and avoid obstacles in real time without prior knowledge of the environment [4].

In the field of LiDAR sensor technology, existing standards, such as DIN SAE SPEC 91471:2023-05 [5] or ASTM E3125-17 Standard [6], provide a robust framework for the assessment of LiDAR sensors irrespective of their type or manufacturing technology. These standards have played a key role in ensuring a uniform evaluation process for LiDAR sensors [7,8]. However, a conspicuous gap in current standards is the absence of a dedicated framework for the evaluation of LiDAR SLAM-based navigation pipelines. This paper aims to contribute to the ongoing discourse by identifying key aspects that should guide future standardization efforts in this area.

To this end, we outline a set of performance indicators that are essential for the objective assessment of SLAM-based navigation using LiDAR technology. Among them, the absolute trajectory error (ATE) remains a fundamental metric for assessing global consistency in pose estimation over time. Beyond accuracy, it is essential to account for the uncertainty associated with the system’s measurements, which reflects the internal confidence of the algorithm and the sensor fusion strategy. Uncertainty evaluation will be described in Section 3. Another critical evaluation parameter is the number of persistently tracked features, which provides an indicator of the SLAM system’s reliability in consistently associating meaningful environmental information. Lastly, computational time must be included as a core evaluation criterion, offering insight into the system’s real-time feasibility, especially for resource-constrained or embedded platforms.

Building on our previous work [9], which explored LiDAR performance within a navigation pipeline, the present study investigates how these evaluation criteria apply when comparing two LiDAR sensors developed with distinct scanning technologies, both integrated into a SLAM-based navigation system for an Unmanned Ground Vehicle (UGV). Specifically, we contrast a LiDAR-SLAM pipeline using a 360° field-of-view sensor based on a motorized optomechanical scanner (the Ouster OS1) with one employing a non-repetitive, limited-field-of-view LiDAR based on MEMS technology (the Livox Horizon). Both systems are evaluated using the state-of-the-art FAST-LIO 2 algorithm [10], chosen for its efficiency, accuracy, and widespread adoption in current research and industry applications, making a robust reference implementation for comparative analysis [11].

Through this experimental comparison, we aim to support the development of a standardized and comprehensive framework for evaluating LiDAR SLAM systems, helping to ensure consistency, interoperability, and performance reliability in real-world autonomous navigation scenarios.

We start by describing the method and experimental setup in Section 3 and Section 4. After that, we present and analyze the results of the experiments in Section 5, and, finally, draw conclusions about the suitability of each sensor type for rover navigation in Section 6.

## 2. Related Works

LiDAR operates on the principle of emitting laser pulses and measuring the time it takes for them to return after hitting an object. This allows for the creation of a 3D map of a vehicle’s surroundings, enabling it to navigate safely. Various techniques, such as time-to-digital converter (TDC), full-waveform, continuous-wave, and photon-counting LiDAR, are being explored to improve range, precision, power consumption, and cost [12]. Technology is rapidly evolving, with a focus on reducing costs and improving performance.

Table 1 illustrates the current LiDAR technologies that rely on Time-of-Flight (ToF) measurements. These technologies include Flash Light LiDAR, OPA scanner LiDAR, MEMS LiDAR, and motorized optomechanical scanners, which offer a range of different features and drawbacks. Flash Light LiDAR technology uses a flash of light to measure distance and has been applied in various fields. Refs. [13,14] both discuss its uses in space exploration, including hazard detection, surface navigation, and autonomous rendezvous and docking. Optical Phased Array (OPA) LiDAR, a non-mechanical beam steering technology, has been the focus of recent research. Refs. [15,16] both highlight the potential of OPA technology in LiDAR applications, with the latter proposing a liquid crystal-tunable OPA for 2D beam steering. MEMS LiDAR relies on Micro-Electro-Mechanical Systems for beam steering, providing compact form factors and low power consumption suitable for integration into small devices [17]. Motorized optomechanical scanners leverage mechanical movements for beam steering, offering high precision and accuracy at the expense of increased complexity and potential mechanical wear [18].

The choice among these LiDAR technologies depends on the specific application requirements, such as range, resolution, speed, and environmental adaptability. Ref. [19] proposes a LiDAR benchmark in realistic drive scenarios, identifying relationships between scan patterns and performance, and providing insights for selecting the best-suited LiDAR for specific applications. Their analysis is focused on the type of target, while, in our case, we focus on the goodness of the trajectory reconstruction and the map. Ref. [20] introduces a simple measurement method for assessing LiDAR performance, exemplified by analyzing the popular VLP 16 by Velodyne. This method enables quick analysis of warm-up time, stability, accuracy of measured coordinates, and laser beam characteristics.

Several studies have been conducted to compare the performance of LiDAR-SLAM techniques for trajectory reconstruction in different scenarios. For example, ref. [21] evaluated the accuracy of the maps created by ROS-based 2D SLAM libraries using a dataset with precise ground truth data. Ref. [22] compared the accuracy and computational requirements of four LiDAR-based SLAM systems for controlling unmanned aerial vehicles (UAVs) in various flight scenarios. Ref. [23] investigated using SLAM for indoor mapping with various sensors. They compared three systems (Matterport, SLAMMER, and NAVIS) in two indoor settings. All three were able to create maps with centimeter-level accuracy. However, the detail and quality of the captured point clouds significantly impacted how well each system suited specific location services. Finally, ref. [24] provides a comparison between three hand-held LiDAR-SLAM systems in an underground environment. To the best of our knowledge, our work is one of the first to compare two different LiDAR technologies in a SLAM scenario using a differential GNSS as the ground truth.

## 3. Method

To carry out a structured and meaningful evaluation of the selected LiDAR-SLAM systems, we defined a set of performance metrics, each targeting a core dimension of SLAM behavior. The methodology is based on acquiring two datasets in distinct outdoor scenarios, with the sensors mounted on board the Unmanned Ground Vehicle, as detailed later in this paper. These evaluation metrics are directly derived from the internal outputs of the FAST-LIO 2 algorithm, which is widely recognized for its high accuracy and computational efficiency [11] and serves as the core framework for our analysis. In particular, FAST-LIO 2 provides both state estimation results and intermediate quantities, such as covariance matrices and feature tracking information, that enable a more granular and interpretable assessment beyond conventional trajectory error indicators. Before introducing and discussing the evaluation metrics, we provide a concise overview of the FAST-LIO 2 algorithm, with a focus on the components relevant to the chosen performance indicators.

### 3.1. FAST-LIO 2 Algorithm Overview

**Figure 1:**
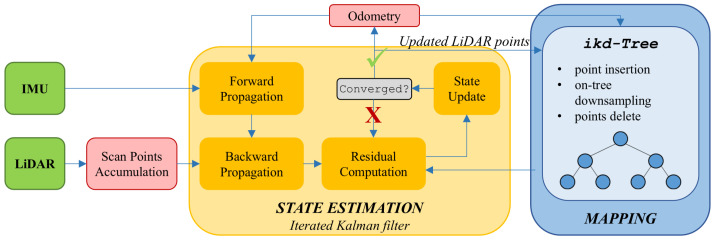
The FAST-LIO 2 pipeline presented in [10]. LiDAR points are accumulated into scans and registered to map points on a large local map via a tightly coupled iterated Kalman filter for state estimation (yellow module). New points are incrementally added to the ikd-Tree for mapping and map management (blue module).

Trajectory estimation is achieved via the FAST-LIO 2 framework [10], which leverages data from both LiDAR sensors and an Inertial Measurement Unit (IMU). A representation of this framework is shown in Figure 1. This method concurrently produces the trajectory of the sensor platform and a detailed point cloud representation of the surrounding environment. The onboard odometry calculation is carried out by fusing LiDAR-derived point cloud information with inertial measurements, employing an iterative Kalman filtering process to enhance accuracy and robustness. FAST-LIO 2 represents a fast, robust, and flexible LiDAR-inertial odometry framework built upon FAST-LIO. Just like in the previous version, FAST-LIO 2 inherits the framework based on the propagation on each IMU measurement and the iterated update on each LiDAR scan to compensate for the motion of every single point.

The algorithm proceeds through a series of steps, starting with the collection of point clouds by the LiDAR devices’ peripherals. Subsequently, preprocessing is performed to remove points detected by the sensor that are outside the field of view. After reducing the number of points and noise in the data using filtering techniques, the LiDAR points are segmented in the field of view.

In parallel with the point accumulation, the state vector representing the pose and the error covariance matrix are initialized and then provided to the Kalman filter. In the prediction step, IMU data are used to forward propagate the state vector in time using the kinematic model, and the error covariance matrix is updated based on the process noise covariance and Jacobian matrix. Subsequently, during the measurement update step, LiDAR data are received and converted to the current estimated pose frame through the measurement model. After combining both steps, the state vector is updated iteratively using the Kalman gain and innovation, resulting in a refined pose.

As Xu et al. showed in [10], to systematically address the increasing computational challenge, FAST-LIO 2 implements a novel data structure, the *ikd-Tree*, which facilitates incremental large local map updates at each step and efficient kNN (k-Nearest Neighbors) queries. Capitalizing on the significantly reduced computational burden, odometry is conducted by directly registering raw LiDAR points onto the map, making the motion tracking process more robust even in cases where detections lack a significant number of relevant features.

### 3.2. Metrics for Evaluation

Based on the outputs made available by the FAST-LIO 2 pipeline, we analyzed four metrics that together form a comprehensive framework for evaluating SLAM performance. These metrics were selected to reflect the most critical system properties for autonomous navigation in real-world environments.

Specifically, we used the ATE as a direct measure of accuracy, allowing us to quantify the deviation between estimated and ground truth poses over time. To assess the system’s internal confidence, we considered the estimation uncertainty, obtained from the covariance matrix of the SLAM state (see point (b) in the following list), which reflects how much trust the system places in its own estimates. As an indicator of reliability, we evaluated the number of persistently tracked features, while the spatial dispersion of these features was assessed qualitatively through visual inspection of the generated maps, to reflect the system’s ability to detect, maintain, and spatially distribute relevant environmental information over time. Finally, the computational time was used as a proxy for efficiency, providing insight into the feasibility of real-time operation, particularly for scenarios with limited hardware or time constraints. Each of these metrics is briefly discussed below:

(a) Absolute Trajectory Error: Pose estimation performances are evaluated in terms of absolute trajectory errors, which are computed for each correspondence of the LiDAR scans as follows:
  (1) $${\mathrm{ATE}}_{i}=\left|{\bar{p}}_{i}-p_{i,\mathrm{GT}}\right|$$
  where ${\bar{p}}_{i}$ is the optimal LiDAR scan position at $t_{i}$ and $p_{i,\mathrm{GT}}$ is the LiDAR ground truth position. For tests in outdoor environments, the most suitable ground truth system is a differential GNSS, which offers centimetric accuracy. In this work, the mean ATE was considered the representative parameter, calculated as the arithmetic average of all ${\mathrm{ATE}}_{i}$ values over the entire trajectory.
(b) Uncertainty: Our uncertainty measure was derived from the covariance matrix in the tangent space of the state estimate, characterized as described in [10]. Suppose that the best state estimate of the most recent LiDAR scan at $t_{i}$ is ${\bar{x}}_{i}$ with a covariance matrix ${\bar{P}}_{i}$. In this case, ${\bar{P}}_{i}$ represents the covariance of the error state vector:
  (2) $${\tilde{x}}_{i}=\left[\delta \theta^{⊤}\;{\tilde{p}}^{⊤}\right]$$
  where δθ=Log(R^⊤WRW) is the attitude error, and the position error $\tilde{p}$ is a standard additive error ($\tilde{p}=p-\hat{p}$). To derive a scalar uncertainty metric, we take the maximum eigenvalue of the covariance matrix, which represents the largest positional dispersion of the rover in that specific case. According to ISO guidelines [25], the standard deviation is the best estimate of the standard uncertainty. Here, we adopt the worst-case condition, given by the square root of the maximum eigenvalue.
(c) Tracked Features: Monitoring the number of tracked features provides valuable information on the effectiveness and efficiency of a LiDAR-SLAM system. This metric reflects the system’s ability to detect and continuously track distinctive features in the environment. A higher number of tracked features generally indicates greater robustness and reliability in pose estimation and mapping.
(d) Computational Time: This metric directly impacts the system’s real-time capabilities. A shorter computational time indicates that the system can rapidly process LiDAR data, extract features, perform data association, and update the map and pose estimation. This is especially critical for applications such as autonomous driving, where timely decisions are essential for safe navigation.

Although not typically defined by a quantitative metric, map quality also plays a relevant role in evaluating SLAM performance. It provides insight into how well the estimated poses align the point cloud to construct a coherent and geometrically accurate representation of the environment. In particular, LiDARs with non-repetitive scanning patterns, such as the Livox Horizon, may benefit from static initialization phases that yield denser and more structured maps. While not listed among the core evaluation criteria, this qualitative aspect is addressed through visual comparison in the discussion, and contributes to the interpretation of the systems’ overall performance.

## 4. Experimental Setup

For the experiment, the MORPHEUS rover (University of Padova, Padova, Italy) [26], a robotic platform currently in development at the University of Padova, was equipped with the two LiDAR sensors under test. These sensors were a $360^{\circ }$ field-of-view Ouster OS1 32 LiDAR (Ouster, San Francisco, CA, USA) sensor and a 3D Livox Horizon LiDAR (Livox, Shenzhen, China), which has a non-repetitive scan pattern and an $81.7^{\circ }$ × $25.1^{\circ }$ FOV. The gyroscope and accelerometer measurements were sampled at 67 Hz by the built-in BMI088 IMU (Bosch, Reutlingen, Germany) module of the Horizon and at 100 Hz by the integrated IAM-20680HT IMU (TDK InvenSense, San Jose, CA, USA) module of the OS1. An overview of the sensor specifications is provided in Table 2.

**Figure 2:**
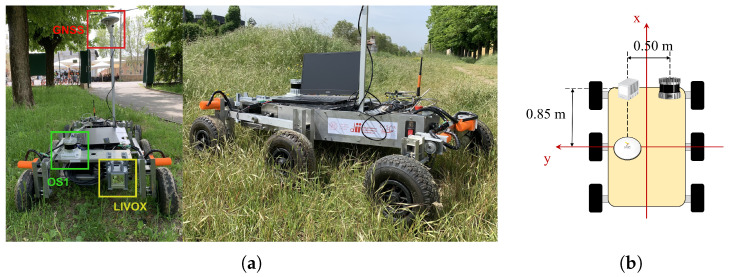
Image of the rover used during the experimental phase. The positions of the OS1-32 Ouster and the Livox Horizon LiDARs are highlighted on the platform. An RTK GNSS receiver was employed as the reference system to provide ground truth data (**a**). The relative position of the sensors with respect to the GNSS antenna is shown in (**b**).

The sensors were placed on board the rover to independently obtain point cloud and inertial measurements. Figure 2 shows the MORPHEUS rover equipped with the Ouster OS1 32 and the Livox Horizon LiDAR, positioned 20 and 30 cm above the ground, respectively. To establish a reliable reference point, an RTK GNSS receiver, the Piksi Multi GNSS Module (Swift Navigation, San Francisco, CA, USA), was employed.

All sensor data were recorded on the same onboard computer, with each sensor managed by an individual Robot Operating System (ROS) node. This configuration ensured precise temporal alignment of all data streams through the system clock, with a single rosbag file captured for analysis. Both LiDAR units internally synchronize their IMU and LiDAR data using built-in microcontrollers and SDKs [27,28], guaranteeing consistent timestamping. Additionally, the FAST-LIO 2 algorithm applies motion compensation by leveraging IMU pre-integration, which refines point cloud accuracy during motion or rotation, thus mitigating potential timing discrepancies.

The data gathered through this approach served as the input for the FAST-LIO 2 algorithm within the ROS, with calculations being performed using a 12th-Generation Intel® Core™ i7-1280P × 20 processor and 16 GB of RAM.

## 5. Results and Discussion

The comparative evaluation of the LiDAR-SLAM systems of two distinct LiDAR technologies, one featuring a motorized optomechanical scanner and the other using an MEMS scanner, revealed some differences in performance.

Data acquisition was carried out in an outdoor environment, moving the MORPHEUS rover along a predefined path, starting from an empty map. Before presenting and analyzing the trajectory reconstructions produced by the SLAM systems, it is essential to provide insight into the reference system used for error computation, given its critical role.

### 5.1. RTK GNSS Validation

**Figure 3:**
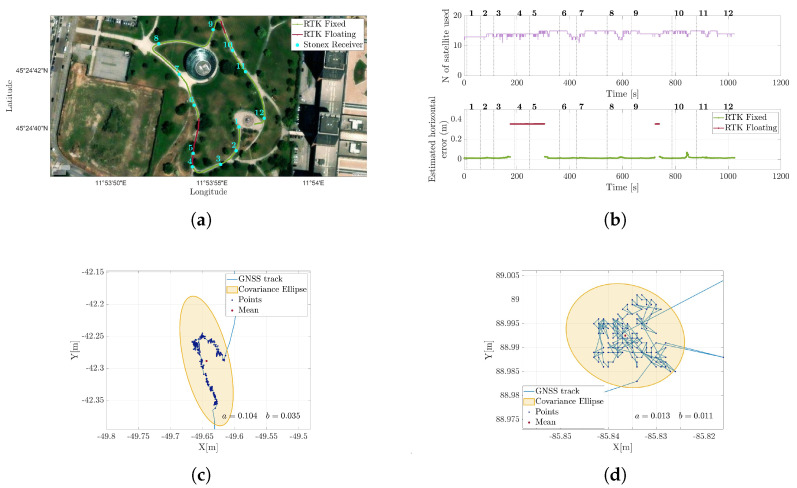
Reconstruction of the trajectory acquired using a Piksi Multi GNSS RTK receiver. (**a**) shows the entire path, color-coded according to the quality of the error estimate. During stops, the position was also recorded using a Stonex receiver, with measurements consistent with those obtained from the differential GPS. (**b**) displays the number of satellites used for position calculation and the estimated horizontal error. (**c**,**d**) show zoomed-in views of Point 4, where RTK correction was not applied, and Point 8, where an RTK fixed solution was obtained, respectively. Superimposed on the trajectory are the covariance ellipses constructed from the points collected during the stops, with a confidence level of 99.7%. In the figures, a and b represent the major and minor semi-axes, respectively.

To validate the reliability of the available differential GPS, a test run was conducted in an open environment similar to that in the upcoming case studies, albeit slightly larger. The path included periodic stops to enrich the characterization of the route and allow for a more in-depth evaluation of GPS performance. Figure 3a illustrates the path followed, with the stopping points clearly marked. Figure 3b presents the number of satellites visible to the system and the estimated horizontal error, provided with 95% confidence [29]. As shown, two distinct performance levels were observed, depending on whether the RTK correction was successfully applied. To better illustrate this difference, Figure 3c,d zoom in on Stop 4—an instance of “RTK float”—and Stop 8—one of several points where full RTK correction was achieved. Covariance ellipses are overlaid for comparison. As is evident, the semi-axes dimensions were significantly smaller when RTK correction was active; the average estimated horizontal error is around 1.5 cm, which meets the requirements of our application. Accordingly, for all the experimental campaigns conducted with the full sensor suite, it was rigorously verified that the GPS measurements included the differential correction throughout the entire acquisition.

### 5.2. Trajectory Reconstruction

**Figure 4:**
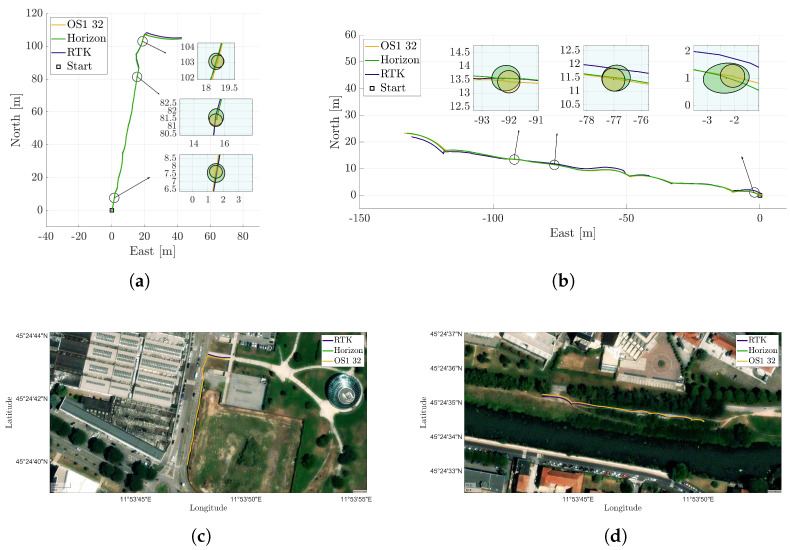
Comparison of the trajectories produced by the LiDAR-SLAM algorithm FAST-LIO 2 utilizing LiDARs in the evaluation phase, namely, the OS1 and Horizon, with the trajectory derived from the RTK reference. (**a**,**b**) represent the metrics of the two trajectories for two different datasets, both overlaid with 99.7% confidence ellipses, derived from the covariance matrix propagated through the Kalman filter. (**c**,**d**) show the superimposition of the three trajectories on a satellite map.

With the reference accuracy validated as sufficient for our needs, two experimental datasets were collected in distinct environments to ensure meaningful comparison. Figure 4a shows the trajectory in a semi-urban setting, consisting of a bike lane segment followed by a park entrance, while Figure 4b refers to a more natural riverside path, characterized by tall grass and sparse surrounding structures. For both cases, LiDAR trajectories reconstructed using the SLAM algorithm FAST-LIO 2 are shown in a top-down view and compared against the RTK-derived reference trajectory. To ensure a consistent reference frame for meaningful comparison, and taking into account the different mounting positions of the sensors on board the rover, each SLAM-estimated trajectory was rigidly aligned to the RTK-GNSS reference frame by applying roto-translation between the two systems. This transformation was accurately estimated through an offline optimization procedure, allowing for precise compensation of the static spatial offset and orientation differences. The cost function minimized in this optimization process can be expressed as follows:

(3) $$\min_{R_{\mathrm{LiDAR}\text{-}\mathrm{GNSS}}^{*},b_{\mathrm{LiDAR}\text{-}\mathrm{GNSS}}^{*}}\sum_{i=1}^{N}\left|\right|t_{\mathrm{GNSS},i}-R_{\mathrm{LiDAR}\text{-}\mathrm{GNSS}}\cdot \left(R_{\mathrm{LiDAR},i}\cdot b_{\mathrm{LiDAR}\text{-}\mathrm{GNSS}}+t_{\mathrm{LiDAR},i}\right){\left|\right|}^{2}$$

where $R_{\mathrm{LiDAR}\text{-}\mathrm{GNSS}}$ is the rotation matrix from the LiDAR frame to the GNSS frame, $b_{\mathrm{LiDAR}\text{-}\mathrm{GNSS}}$ is the static offset between the LiDAR and GNSS sensors, $R_{\mathrm{LiDAR},i}$ and $t_{\mathrm{LiDAR},i}$ are the rotation and position of the LiDAR at time *i* estimated by LiDAR-SLAM, and $t_{\mathrm{GNSS},i}$ is the GNSS trajectory at time *i*. The optimization aims to find the optimal $R_{\mathrm{LiDAR}\text{-}\mathrm{GNSS}}^{*}$ and $b_{\mathrm{LiDAR}\text{-}\mathrm{GNSS}}^{*}$ that minimize the difference between the transformed LiDAR trajectory and the GNSS trajectory over all *N* time steps. As an initial condition for the optimization process, we used the baseline measured manually with a ruler, as visible in Figure 2b, providing a reasonable starting point for $b_{\mathrm{LiDAR}\text{-}\mathrm{GNSS}}$.

**Figure 5:**
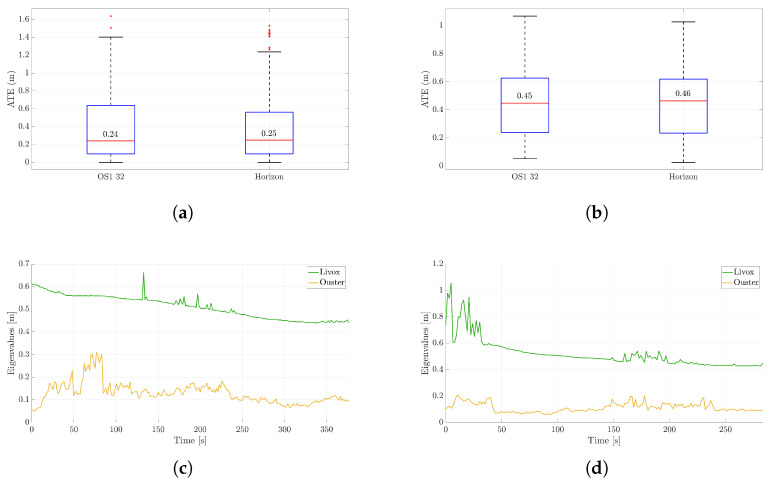
Metrological performances of the two sensors, namely, the OS1 and Horizon LiDARs. (**a**,**c**) refer to the scenario represented in Figure 4a, while (**b**,**d**) correspond to the one shown in Figure 4b. In (**a**,**b**), the absolute trajectory error of the trajectories estimated by the LiDAR-SLAM algorithm FAST-LIO 2 is given with respect to the RTK reference. Furthermore, the maximum of the eigenvalues of the covariance matrix propagated through the Kalman filter is illustrated in (**c**,**d**).

Figure 5a,b illustrate the ATE for both LiDAR trajectories in the two different scenarios, displaying average values of 0.25 m and 0.46 m for the Livox Horizon for the first and second case, respectively, and 0.24 m and 0.45 m for the Ouster, computed as described in point (a) of Section 3.2. Although one might expect the Ouster to perform better due to its larger field of view, the trajectory estimation results for the two LiDARs are comparable. This outcome is likely due to a combination of factors (see Table 2):

- The random error of the Horizon is lower in distance measurement;
- The gyroscope resolution of the Horizon is greater than that of the OS1;
- The non-repetitive pattern of the Horizon results in a more densely populated point cloud within its field of view.
- The Livox FOV is flatter, but it extends further in depth. This allows for collecting points at greater distances and thus enables better rotation estimation.

Parallel to the trajectory reconstruction, an uncertainty analysis was carried out. In Figure 4a,b, the projection onto the plane of uncertainty ellipses is shown at three different locations on the map. Figure 5c,d illustrate the evolution of the maximum eigenvalues of the 3D covariance matrix at each step, a representative index of the uncertainty in the rover position coming from the Kalman filter in FAST-LIO 2 and the sensors along with their positions. According to [15], the values are provided with a 99.7% confidence level.

Showcasing the filter’s convergence, there is a gradual reduction in uncertainty for both curves. Furthermore, it is apparent that the estimated uncertainty in the position of the Livox LiDAR is higher than that of the Ouster LiDAR. This discrepancy arises from the Ouster LiDAR’s advantage of having a greater number of features that are more evenly distributed around the rover. Conversely, the Livox LiDAR’s constrained field of view limits the number of features available for tracking, leading to increased uncertainty in its position estimation.

For the following discussion, to avoid redundancy and enhance clarity, only the first scenario, corresponding to a lower ATE, will be developed.

### 5.3. Points Acquisition and Computational Time Comparison

**Figure 6:**
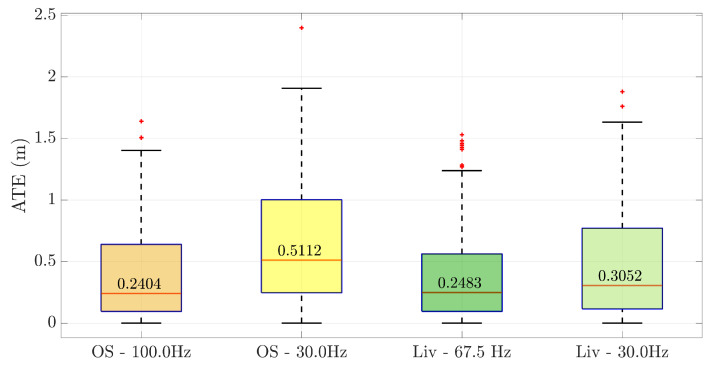
ATE of the scenario represented in Figure 4a as the IMU sampling frequency varies.

The effect of the IMU update frequency on trajectory reconstruction has been analyzed. Specifically, the acquisition rate was set to 30 Hz for both the IMUs. In Figure 6, it is possible to see the trend of the ATE for the cases considered. Synchronizing the acquisition frequency of both IMUs supported the earlier observations. Indeed, the Horizon, benefiting from a wider range and lower noise, shows a reduced error.

**Figure 7:**
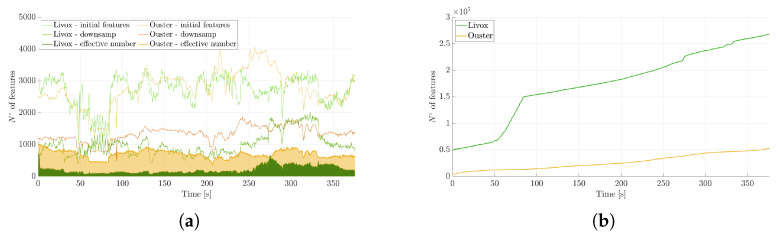
LiDAR feature screening. (**a**) illustrates the screening of points acquired by the two LiDARs, while (**b**) shows the increase in map features over time.

Figure 7a illustrates the screening of points acquired by the two LiDARs, and the final number is marked as the ”effective number”. This count specifically refers to points for which a sufficient number of nearby points can be located to enable the fitting of a small plane patch, facilitating the determination of the normal used in pose estimation within the measurement model. As is visible in the figure, the Ouster LiDAR has a higher number of effective points compared to the Livox Horizon. This is the feature that allows the Ouster to achieve slightly better performance in the trajectory reconstruction, although, as already emphasized, this is not the only aspect to consider.

Another interesting aspect to highlight is the transition from the first to the second curve (named ”initial features” and ”downsamp”, respectively) for each sensor. Given that the Livox LiDAR has a denser point cloud, the effect of the sampling transition is more pronounced for this sensor.

Simultaneously, a map was constructed and maintained, storing points following the hierarchical data structure provided by the k-d tree, enabling efficient searching of nearest points in three-dimensional space. As is visible in Figure 7b, the Livox LiDAR gradually builds a denser map over time, even if the effective number of points used by FAST-LIO 2 for pose estimation is significantly higher for the Ouster LiDAR, which takes advantage of its wider field of view.

**Figure 8:**
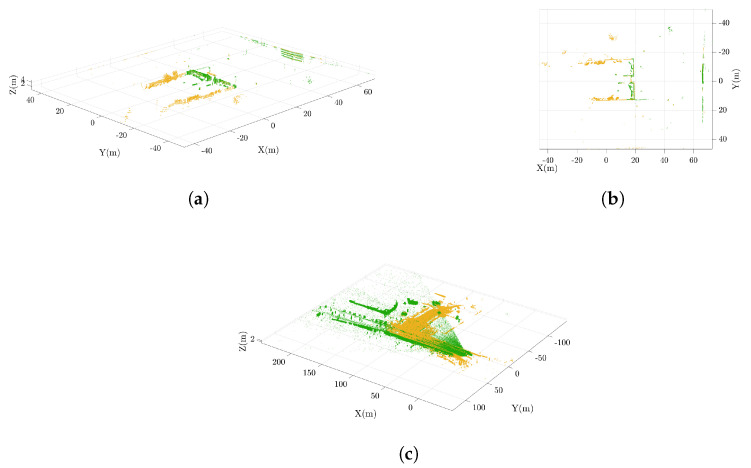
Point clouds from the OS1 (yellow) and Horizon (green) sensors. Subfigures (**a**,**b**) show the comparison between the two point clouds acquired in a single frame from two different points of view, while (**c**) represents the entire point clouds collected before the downsampling step.

**Figure 9:**
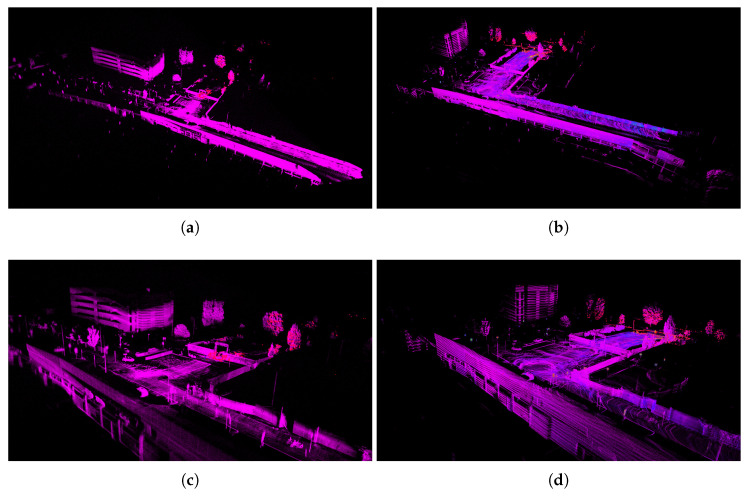
Visualization of the maps in Rviz, a 3D visualization tool for ROSs. Panels (**a**,**b**) show the final maps obtained with the Livox Horizon and Ouster LiDAR sensors, respectively, while panels (**c**,**d**) provide a closer view of the corresponding map details, highlighting the differences in feature representation between the two sensors.

This result is once again explained by the fact that the Livox LiDAR captures denser point clouds. In Figure 8c and Figure 9a,b, it is possible to observe the difference in the constructed maps. While the Ouster even gathers features behind itself, as seen by comparing the aforementioned images in the bottom right, the Horizon LiDAR collects more points (see details in Figure 9c,d) and maintains a deeper field of view, in accordance with the specifications provided in Table 2. In Figure 8a,b, the overlap of the point clouds from the two sensors at the same instant is illustrated. As previously pointed out, the arrangement of points clearly highlights the diversity of ranges, cloud density, and fields of view.

Regarding performance, another comparison is made for FAST-LIO 2 computational aspects. Each time step includes the following stages:

- Preprocessing of LiDAR data (including IMU data), filtering, downsampling, and map segmentation within the LiDAR’s FoV;
- Matching current keypoints with previous ones, solving the Iterative Closest Point (ICP) algorithm, and iterating the Kalman filter update;
- Map incrementation.

**Figure 10:**
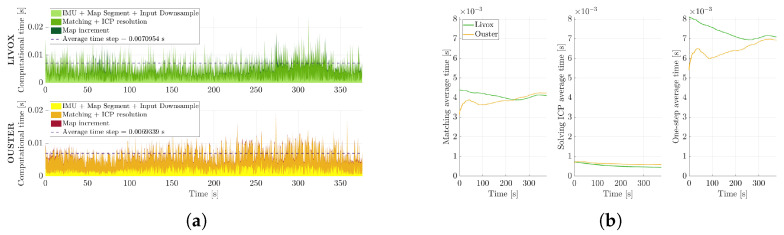
Computational time. (**a**,**b**) illustrate the screening of computational time steps over time.

Figure 10a represents the time steps divided as described above, highlighting the final average time step, which shows a slightly lower value for the Ouster. This result appears to be in disagreement with the first two images in Figure 10b, which show lower average match and solve times for the Livox Horizon. However, the most time-consuming part coincides with the map incrementation step; since the Horizon LiDAR collects more points, it is expected that the map update step may be slightly longer.

To summarize, Table 3 reports the comparison of the two sensors based on the main evaluation metrics described above. As a quantitative indicator of reliability, we considered the number of persistently tracked features, as detailed in the previous section. However, despite notable differences in point count, the tested scenarios did not reveal a clear predominance of reliability for either system. This is because a full assessment of reliability also requires taking into account other aspects, such as the field of view width and depth, as well as the spatial distribution of features. The Livox sensor provides a deeper but narrower scan, while the Ouster offers a wider field of view with more uniformly distributed features. This more homogeneous spatial coverage slightly reduces pose uncertainty, as the features are better spread across the scene and more effective for motion estimation. Conversely, the Livox compensates through its deeper range perception and lower random noise, ultimately achieving comparable trajectory accuracy. Lastly, due to its higher number of detected features, the Livox requires slightly more computation time, although still within real-time constraints.

## 6. Conclusions

In this paper, the key metrics essential for evaluating a LiDAR-SLAM system have been identified. These include the absolute trajectory error, uncertainty, the number of persistently tracked features, and the computational time required for pose updates. The results emphasize that the accuracy of a LiDAR-SLAM system is not solely determined by the robustness of the algorithm or the metrological performance of the sensor individually, but rather by the synergy between both components. To validate this, we evaluated and compared two LiDAR technologies integrated into a SLAM pipeline: the Ouster OS1, a motorized optomechanical LiDAR with a 360° field of view, and the Livox Horizon, a lower-cost MEMS-based sensor with a narrower, non-repetitive scan pattern.

Despite substantial differences in field of view, detection range, noise, and cost, both systems demonstrated comparable localization performance. The estimated trajectory errors were remarkably similar, with the OS1 achieving an average ATE of 0.24 m and the Horizon achieving 0.25 m. These results indicate that both LiDARs, when used with a high-performance algorithm such as FAST-LIO 2, can yield accurate and reliable trajectory reconstruction.

Further analysis revealed trade-offs in computation and mapping characteristics. The Livox Horizon tends to accumulate more points in the local map, increasing computation time due to the larger number of updates required. However, the OS1’s broader and more uniform field of view provides a better spatial distribution of features, resulting in a greater number of effective points for pose estimation and slightly more stable performance.

While the OS1 showed marginally better reliability and consistency in pose estimation, the differences were not statistically significant. Importantly, the Livox Horizon proved capable of delivering similar SLAM performance despite its reduced field of view and lower cost. These findings suggest that, for many applications, particularly those with budget or payload constraints, MEMS-based LiDAR sensors like the Livox Horizon can serve as effective alternatives to more expensive full-view LiDARs, without compromising localization accuracy.

To further strengthen the comparison, future studies should include a broader range of testing environments, particularly those with varying terrain types and structural complexity, to evaluate system robustness across more diverse real-world conditions. Moreover, this analysis relied exclusively on the FAST-LIO 2 SLAM algorithm, which is among the most accurate and efficient state-of-the-art methods currently available. An important extension of this work will therefore be to compare the results with those obtained using other high-performing SLAM frameworks, in order to better assess the generalizability of the observed trends and the interplay between hardware and algorithmic design.

Overall, the study highlights that selecting a SLAM configuration should not rely solely on sensor specifications or algorithmic reputation, but on a holistic evaluation of their interplay within the intended operational context.

## Figures and Tables

**Table 1:** Existing LiDAR technologies based on ToF measurements.

Technology	Type	Description	Manufacturers
Flash Light	Non-scanning LiDAR	An array of photodetectors at the image plane captures the Time-of-Flight data of each pixel in the two-dimensional field of view.	${\text{LeddarTech }}^{\left(1\right)}$, ${\text{Ouster }}^{\left(2\right)}$ (Digital Flash)
OPA Scanners	Scanning LiDAR (non-mechanical scanners)	The laser power is divided into a collection of transmitters, each of which can be adjusted in terms of phase. By adjusting the relative phase shifts among the transmitters, it is possible to create and direct a laser beam.	${\text{Lumotive }}^{\left(3\right)}$
MEMS Scanners	Scanning LiDAR (mechanical scanners)	MEMS mirrors have the ability to direct, modulate, and switch light, in addition to controlling phase.	${\text{Innoviz }}^{\left(4\right)}$, ${\text{Livox }}^{\left(5\right)}$
Motorized Optomechanical Scanners	Scanning LiDAR (mechanical scanners)	The beam is steered by one or more rotating parts.	${\text{Velodyne }}^{\left(6\right)}$, ${\text{Ouster }}^{\left(2\right)}$ (OS)

^(1)^ LeddarTech Holdings Inc., Québec, QC, Canada; ^(2)^ Ouster, San Francisco, CA, USA; ^(3)^ Lumotive, Redmond, WA, USA; ^(4)^ Innoviz Technologies Ltd., Rosh HaAyin, Israel; ^(5)^ Livox, Shenzhen, China; ^(6)^ Merged with Ouster.

**Table 2:** LiDAR specifications as stated by the manufacturers’ datasheets.

	Livox Horizon	Ouster OS1 32 Channel
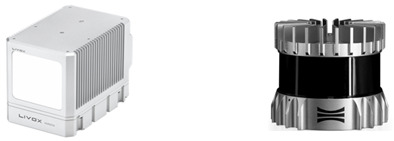		
Maximum Detection Range	90 m @ reflectivity 10%	90 m @ reflectivity 10%
	130 m @ reflectivity 20%	170 m @ reflectivity 80%
	260 m @ reflectivity 80%	
Minimum Detection Range	0.5 m	0.5 m
FOV (H×V)	81.7° × 25.1°	360° × 45°
FOV ${\text{Coverage}}^{1}$	60% @ 0.1 s	${\text{NA}}^{1}$
	98% at 0.5 s	
Point Acquisition Rate	>240,000 points/s	1,310,720 points/s
Random Error (1−σ)	0.02 m @ 20 m	0.05 m @ 0.5 m
		0.1 m @ 90 m
	**IMU BMI0881**	**IMU IAM-20680HT**
Resolution	Accelerometer (A): 0.09 mg	Accelerometer (A): 0.06 mg
	Gyroscope (G): 0.004°/s	Gyroscope (G): 0.008°/s
Measurement Range	(A) ±3 g	(A) ±2 g
	(G): ±125°/s	(G): ±250°/s
Noise Density (typ.)	(A): 175 μg/$\sqrt{\mathrm{Hz}}$	(A): 135 μg/$\sqrt{\mathrm{Hz}}$
	(G): 0.014°/s/$\sqrt{\mathrm{Hz}}$	(G): 0.005°/s/$\sqrt{\mathrm{Hz}}$
Sample Rate	67 Hz	100 Hz

^1^ Only applicable for Horizon as it is a non-repetitive scanning LiDAR.

**Table 3:** Final summary of the comparison between the two LiDARs. + and +++ are used to indicate a better or worse result, respectively, while ++ denotes equivalence.

	Livox Horizon	Ouster OS1
ATE error	++	++
Uncertainty	+	+++
Tracked features	+++	+
Computational time	+	+++

## Data Availability

The original contributions presented in this study are included in the article. Further inquiries can be directed to the corresponding author.

## Abbreviations

The following abbreviations are used in this manuscript:

ATE	Absolute Trajectory Error
GNSS	Global Navigation Satellite System
GPS	Global Positioning System
LiDAR	Light Detection And Ranging
MEMS	Micro-Electro-Mechanical Systems
OPS	Optical Phased Array
ROS	Robot Operating System
RTK	Real-Time Kinematic
SLAM	Simultaneous Localization And Mapping
ToF	Time of Flight
